# *Bacteroidota* and *Lachnospiraceae* integration into the gut microbiome at key time points in early life are linked to infant neurodevelopment

**DOI:** 10.1080/19490976.2021.1997560

**Published:** 2021-11-28

**Authors:** Kaitlyn Oliphant, Mehneez Ali, Mark D’Souza, Patrick D. Hughes, Dinanath Sulakhe, Annie Z. Wang, Bingqing Xie, Rummanu Yeasin, Michael E. Msall, Bree Andrews, Erika C. Claud

**Affiliations:** a Department of Pediatrics, Biological Sciences Division, University of Chicago, Chicago, IL, USA; b Center for Research Informatics, Biological Sciences Division, University of Chicago, Chicago, IL, USA; c Department of Pediatrics, Division of Neonatology, NorthShore University HealthSystem, Evanston, IL, USA; d Department of Medicine, Biological Sciences Division, University of Chicago, Chicago, IL, USA; e Kennedy Research Center on Intellectual and Developmental Disabilities, University of Chicago, Chicago, IL, USA

**Keywords:** Human gut microbiome, infant microbiome succession, infant neurodevelopment, dispersal limitation, habitat filtering

## Abstract

The early life microbiome plays critical roles in host development, shaping long-term outcomes including brain functioning. It is not known which initial infant colonizers elicit optimal neurodevelopment; thus, this study investigated the association between gut microbiome succession from the first week of life and head circumference growth (HCG), the earliest validated marker for neurodevelopment. Fecal samples were collected weekly from a preterm infant cohort during their neonatal intensive care unit stay and subjected to 16S rRNA gene sequencing for evaluating gut microbiome composition, in conjunction with clinical data and head circumference measurements. Preterm infants with suboptimal HCG trajectories had a depletion in the abundance/prevalence of *Bacteroidota* and *Lachnospiraceae*, independent of morbidity and caloric restriction. The severity of gut microbiome depletion matched the timing of significant HCG pattern separation between study groups at 30-week postmenstrual age demonstrating a potential mediating relationship resultant from clinical practices. Consideration of the clinical variables indicated that optimal infant microbiome succession is primarily driven by dispersal limitation (i.e., delivery mode) and secondarily by habitat filtering (i.e., antibiotics and enteral feeding). *Bacteroidota* and *Lachnospiraceae* are known core taxa of the adult microbiome, with roles in dietary glycan foraging, beneficial metabolite production and immunity, and our work provides evidence that their integration into the gut microbiome needs to occur early for optimal neurodevelopment.

## Introduction

The first five years of life are widely recognized to be critical for an individual’s educational and vocational success,^1^ as well as lifelong health and wellbeing^2^. On a cellular neurodevelopmental level, synaptogenesis and myelination occur at a net positive during this timeframe increasing brain volume until 90% of adult size is reached by age five.^3,4^ These processes are known to be both programmed by genetics and responsive to environmental cues,^5^ and the consequence of their impediment is developmental disability, a group of conditions resultant from physical, learning, language, or behavioral impairment that often lasts throughout an individual’s lifetime.^6^ Developmental disability was estimated by the Global Burden Disease Study 2016 to affect 8.4% of children under five years of age worldwide,^7^ and although global initiatives specifically targeting this timeframe for improving overall human health and economics have achieved success in reducing child mortality,^8^ the rate of developmental disability remains minimally changed.^7^ Thus, there is a vital need to identify modifiable environmental factors to reduce the incidence of developmental disabilities. Infancy represents a potentially crucial stage for intervention, as diagnosis of these developmental disabilities can already occur by 2–3 years of age.^9,10^ An early marker of neurodevelopment is necessary, and head circumference growth (HCG) has been found to sufficiently proxy increase in brain volume, thus correlating well with later neurodevelopmental testing results.^11–15^

Studies to date have focused on nutritional interventions for improving HCG in infants.^16–20^ However, associations between infant diet and neurodevelopment are not always clear; for example, a recent meta-analysis on human milk feeding for improving neurodevelopmental outcomes demonstrated only weak or inconclusive evidence for its beneficial effects. One overlooked factor that is gaining attention is the ecosystem of microorganisms that populate the gastrointestinal tract, the gut microbiome. The gut microbiome diversifies in parallel with infant development,^21–24^ and antibiotic use in the first year of life has been correlated with later developmental outcomes, including lower cognitive capabilities, attention deficit hyperactivity disorder (ADHD), anxiety and depression.^25,26^ The gut microbiome also intersects with many environmental factors already associated with neurodevelopment,^27–30^ importantly including nutrition. Critical roles of the gut microbiome in digestion include breaking-down macronutrients, producing neuroactive metabolites after fermentation, and synthesizing micronutrients.^5,31^ The gut microbiome additionally indirectly affects neurodevelopment via its interactions with the immune system and intestinal barrier, impacting systemic inflammation and circulation of metabolites.^31^ Alterations in the composition and function of the gut microbiome have been associated with developmental disabilities in children including autism spectrum disorder (ASD),^32^ and ADHD.^33^ However, it is not known which early life microbes occurring prior to developmental disability onset are vital to optimal neurodevelopment, and no study to date has examined the relationship between the earliest neurodevelopmental marker, HCG, and the gut microbiome in infants over time.

To address this knowledge gap, prospectively collected longitudinal fecal samples from 58 infants born <34 weeks gestational age at the University of Chicago Comer Children’s Hospital were subjected to 16S rRNA gene sequencing,^34,35^ to determine the microbiome composition and predicted functional profile.^36^ Preterm infants were selected as they represent a substantive cohort (1 in every 10 infants worldwide is born preterm^37^) at an increased risk for both developmental disability,^38^ and altered gut microbiome configurations from clinical exposures such as antibiotics.^21–24^ Further, preterm infants can be strictly monitored within the neonatal intensive care unit (NICU) environment, allowing statistical and machine learning models to be built that incorporate HCG trajectories, time, and clinical variables. We hypothesized that features of the early gut microbiome are associated with HCG from birth to term-equivalent age in the NICU. It was found that infants with suboptimal HCG trajectories (SHCGT) experienced a concurrent loss in HCG and the abundances of *Bacteroidota* and *Lachnospiraceae* specifically from 31 to 36 weeks postmenstrual age (PMA), indicating a mediating effect by these microbial taxa on HCG that was independent of clinical morbidity and caloric restriction but likely resultant from preceding clinical practices. Thorough examination of the potentially casual clinical variables revealed preferential effects of infant microbiome successional drivers, with dispersal limitation (i.e., delivery mode) superseding habitat filtering (i.e., antibiotics and enteral feeding) in shaping optimum microbiome and thus host development.

## Results

### Gut microbiome maturation and infant head circumference growth trajectory divergence exactly coincide at 30 weeks postmenstrual age, demonstrating that the infant gut microbiome is an associated mediator of neurodevelopment

**Figure 1.**
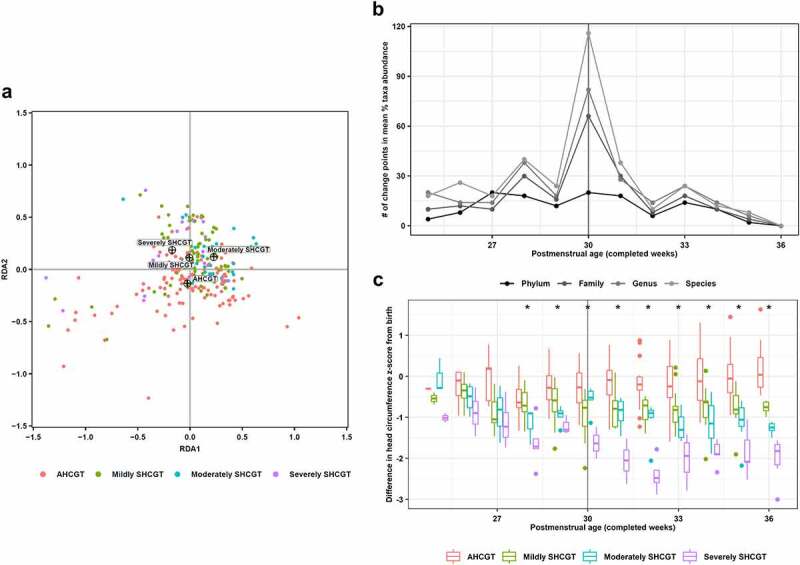
Changes in infant head circumference growth and gut microbiome β-diversity over completed weeks postmenstrual age. Head circumference growth group is defined by the difference in head circumference z-score from birth to 36 weeks postmenstrual age as calculated by the Fenton growth curve: appropriate head circumference growth trajectory (≥0.5; AHCGT), mildly suboptimal head circumference growth trajectory (<0.5–1; mildly SHCGT), moderately suboptimal head circumference growth trajectory (<1-1.5; moderately SHCGT) and severely suboptimal head circumference growth trajectory (<1.5; severely SHCGT). (a) Redundancy analysis of 16S rRNA gene sequencing data generated from fecal samples collected weekly. Samples are colored by head circumference growth group, with the centroids for each group indicated by crosshairs. (b) Number of change points, as identified by non-parametric analysis, in mean percent abundance pattern of each microbial taxon by head circumference growth group for each completed postmenstrual age week. Data are shown for all examined taxonomic levels from phylum to species. (c) Difference in head circumference z-score from birth as calculated by the Fenton growth curve for each completed postmenstrual age week by head circumference growth group. Box-plot center line, median; limits, first and third quartiles; whiskers, 1.5× interquartile range; points, outliers. *p< .05, Welch’s ANOVA

It was not previously known if gut microbiome succession could be linked to HCG, the earliest marker of human neurodevelopment. This study not only determined that β-diversity of the gut microbiome significantly differentiated infants with SHCGT from appropriate HCG trajectories (AHCGT) (Figure 1(a)), but also that the key time point when most fecal microbial taxa exhibited a shift in mean abundance of 30 weeks PMA (Figure 1(b)) matched the onset of significant SHCGT (Figure 1(c)). The matched timing suggests that preceding clinical practices may impact gut microbiome succession which changes infant HCG trajectories; this relationship is defined as mediation and the interactions between these three factors (clinical variables, the gut microbiome and infant HCG) will thus be examined in further detail. Preterm infant HCG trajectory was assessed as the difference in head circumference z-score from birth to 36 weeks PMA by the Fenton growth curve,^14,39^ and study groups were stratified by 0.5 interval losses in z-score: AHCGT (≥0.5), mildly SHCGT (<0.5–1), moderately SHCGT (<1-1.5), and severely SHCGT (<1.5). Alterations in α-diversity between study groups were first considered using ANOVA on multivariate regression with PMA as a covariate and patient as a random effect, but no significant differences were found (Table S1). Next, β-diversity (genus level) was examined by redundancy analysis with study groups and PMA as terms and permutations blocked by patient (Figure 1(a)). A significant redundancy analysis model was built (*p*= .001), and all terms had a statistically significant effect on gut microbiome composition (*p*= .001), listed from most to least impact (R^2^): PMA (0.6391), AHCGT (0.3795), moderately SHCGT (0.2160), severely SHCGT (0.1447), and mildly SHCGT (0.1060).

Defining key time points of gut microbiome succession is critical, as previous research has shown that the inability of the microbiome to achieve a particular state on time (i.e., “mature”) is associated with poor developmental outcomes such as weight gain failure in children.^40^ To identify successional time points within the infant dataset, a novel exploitation of change point analysis was employed (see methods section for details). After application of this approach, 30 weeks PMA was determined to be the key time point of gut microbiome succession within our studied time range, regardless of taxonomic level (Figure 1(b)). To observe how this key time point correlated with HCG patterns, the loss in head circumference z-score from birth for each PMA week was plotted for each study group (Figure 1(c)). ANOVA with post-hoc analysis revealed that the severely SHCGT group significantly separated from the AHCGT group starting at 28 weeks PMA, but significantly differentiating all three SHCGT groups from AHCGT could not be done until 31 weeks PMA (Table S2). Additionally, all three SHCGT groups could not be significantly distinguished from each other until the measurement end point of 36 weeks PMA (Table S2). Together, these results indicate that SHCGT could generally be treated as a loss in head circumference z-score of greater than 0.5 that occurred from 31 to 36 weeks PMA. Therefore, concordance of the gut microbiome successional pattern with infant HCG indicates a potential mediating role for an “immature” microbiome resultant from preceding clinical practices in promoting SHCGT, and further analysis of the infant gut microbiome was thus conducted on the time windows of 24–30 weeks PMA (before SHCGT) and 31–36 weeks PMA (during SHCGT), as well as with consideration of the clinical variables.

### Infants with suboptimal head circumference growth trajectories have a depleted abundance of fecal Bacteroidota and Lachnospiraceae from 31 to 36 weeks PMA

**Figure 2.**
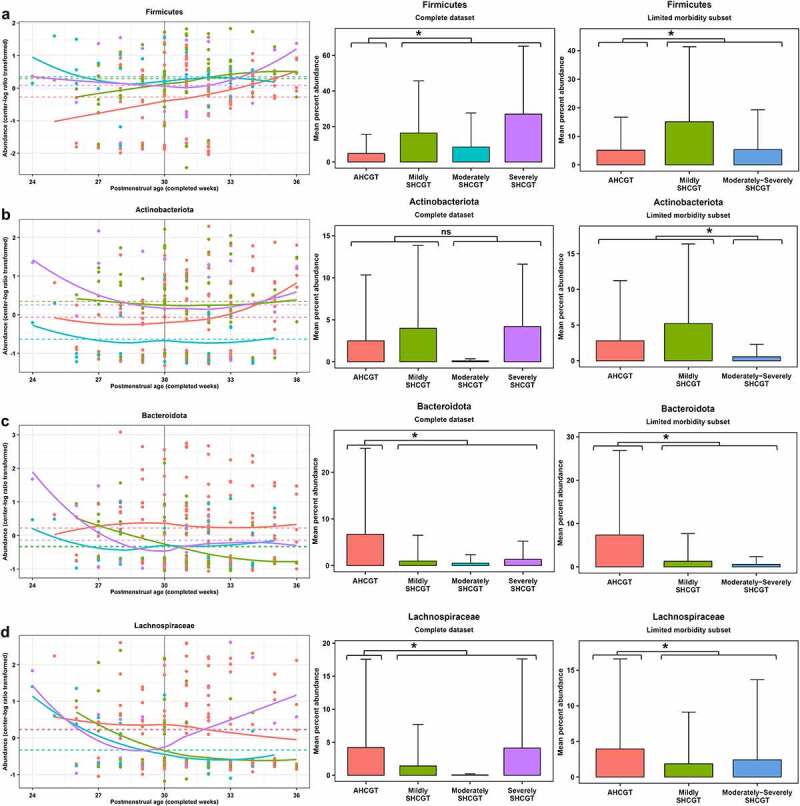
Abundance of fecal microbial taxa that significantly differ by infant head circumference growth. Head circumference growth groups are defined in legend for Figure 1. Left panel displays center-log ratio transformed abundance pattern over completed weeks postmenstrual age for the complete dataset, with the solid line indicating the LOESS regression result for each study group and the dashed line indicating the least squares mean for each study group from linear mixed regression including postmenstrual age as an additional fixed effect and patient as a random effect. Middle and right panels display mean percent abundance with standard deviation bars for both the complete and limited morbidity datasets, respectively. *p< .05; FP<1%, ANOVA on multivariate regression. (a) *Firmicutes*. (b) *Actinobacteriota*. (c) *Bacteroidota*. (d) *Lachnospiraceae.*

Having identified the time point of microbiome change relevant to head circumference growth, we next investigated which microbial taxa are putatively important for infant neurodevelopment. *Bacteroidota* (Figure 2(c)) and *Lachnospiraceae* (Figure 2(d)) were specifically identified as potential biomarkers of AHCGT. Further, it was determined that their significant reduction in abundance occurred during the time period of head circumference z-score loss (31–36 weeks PMA) (Figure 1(c)), solidifying a presumed mediating relationship and a promising key time point for interventional strategies. Finally, through predictive metagenomic profiling,^36,41^ the depletion of these taxa was found to potentially result in a reduced carbohydrate utilization capacity of the gut microbiome (Table 1), suggesting a mechanistic link in regards to energy resource utilization and short-chain fatty acid production.^5,31^ The differences in microbial taxon and KEGG orthology (KO) abundances between study groups were assessed by ANOVA on multivariate regression with patient as a random effect during the time windows of 24–30 weeks PMA (before SHCGT) and 31–36 weeks PMA (during SHCGT). The number of patients with microbial taxon or KO presence versus absence for each study group was statistically compared by the Fisher’s exact test. Study group comparisons also included infants with any SHCGT versus AHCGT, and infants with moderately SHCGT to severely SHCGT versus AHCGT to mildly SHCGT. However, the latter yielded no statistically significant differences in abundance or prevalence of microbial taxa, and thus will not be discussed further.

**Table 1.** Metabolic KEGG pathways containing the most significantly differentially abundant KOs by infant head circumference growth

KEGG Pathway	AHCGT vs. SHCGT
1. CARBOHYDRATE METABOLISM	16
1.1 Starch and sucrose metabolism:	8
*glycogen synthase [EC:2.4.1.11]*	Overall: *p*= .0006, diff = 0.57 [0.26, 0.89], R^2^ = 0.10; *p*= ns, 75% vs. 60%
	31–36: *p*= .00007, diff = 5.12 [2.80, 7.44], R^2^ = 0.14; *p*= .004, 72% vs. 28%
*glucan 1,4-α-glucosidase [EC:3.2.1.3]*	Overall: *p*= .001, diff = 0.55 [0.22, 0.87], R^2^ = 0.10; *p*= ns, 86% vs. 77%
	31–36: *p*= .0003, diff = 5.46 [2.70, 8.22], R^2^ = 0.14; *p*= ns, 80% vs. 44%
*glycogen phosphorylase/synthase [EC:2.4.1.1 2.4.1.11]*	Overall: *p*= .0004, diff = 0.58 [0.27, 0.89], R^2^ = 0.10; *p*= ns, 75% vs. 63%
	31–36: *p*= .0001, diff = 5.16 [2.70, 7.61], R^2^ = 0.13; *p*= .004, 72% vs. 28%
*α-amylase [EC:3.2.1.1]*	Overall: *p*= .009, diff = 0.45 [0.12, 0.79], R^2^ = 0.07; *p*= ns, 79% vs. 67%
	31–36: *p*= .005, diff = 4.24 [1.38, 7.10], R^2^ = 0.09; *p*= .009, 80% vs. 40%
*hexokinase [EC:2.7.1.1]*	Overall: *p*= .003, diff = 0.52 [0.18, 0.86], R^2^ = 0.07; *p*= ns, 64% vs. 37%
	31–36: *p*= .002, diff = 3.36 [1.41, 5.32], R^2^ = 0.09; *p*= .007, 56% vs. 16%
1.2 Amino sugar and nucleotide sugar metabolism:	5
*fucokinase [EC:2.7.1.52]*	Overall: *p*= .001, diff = 0.54 [0.22, 0.86], R^2^ = 0.09; *p*= ns, 75% vs. 57%
	31–36: *p*= .0005, diff = 4.54 [2.12, 6.96], R^2^ = 0.12; *p*= .002, 72% vs. 24%
*N-acylglucosamine 2-epimerase [EC:5.1.3.8]*	Overall: *p*= .004, diff = 0.49 [0.16, 0.82], R^2^ = 0.08; *p*= ns, 79% vs. 67%
	31–36: *p*= .003, diff = 4.48 [1.59, 7.37], R^2^ = 0.10; *p*= .009, 80% vs. 40%
*N-acetyl-α-D-muramate 1-phosphate uridylyltransferase [EC:2.7.7.99]*	Overall: *p*= .005, diff = 0.43 [0.13, 0.72], R^2^ = 0.04; *p*= ns, 100% vs. 100%
	31–36: *p*= .002, diff = 2.68 [1.03, 4.34], R^2^ = 0.11; *p*= ns, 100% vs. 92%
*mannose-1-phosphate guanylyltransferase [EC:2.7.7.13]*	Overall: *p*= .01, diff = 0.47 [0.12, 0.82], R^2^ = 0.06; *p*= ns, 100% vs. 100%
	31–36: *p*= ns, diff = 0.94 [0.11, 1.77], R^2^ = 0.04; *p*= ns, 100% vs. 100%
1.3 Fructose and mannose metabolism:	3
1.4 Butanoate metabolism:	3
2. GLYCAN BIOSYNTHESIS AND METABOLISM	11
3. METABOLISM OF COFACTORS AND VITAMINS	9
4. LIPID METABOLISM	6
4. ENERGY METABOLISM	6
7. AMINO ACID METABOLISM	5
8. BIOSYNTHESIS OF OTHER SECONDARY METABOLITES	4
9. NUCLEOTIDE METABOLISM	3
9. METABOLISM OF TERPENOIDS AND POLYKETIDES	3

Legend: Study groups defined by difference in head circumference z-score from birth to 36 weeks postmenstrual age as calculated by the Fenton growth curve: appropriate head circumference growth trajectory (≥0.5; AHCGT) and suboptimal head circumference growth trajectory (<0.5; SHCGT). For KEGG pathway classifications, the total number of significantly differentially abundant or prevalent KEGG database orthologies (KOs) is indicated with at least 3 being requisite for listing. Significance for abundance was evaluated by ANOVA on multivariate regression with infant head circumference growth trajectory and postmenstrual age as fixed effects and patient as a random effect, and for prevalence by the Fisher’s exact test (*p* < 0.05; FP<1%). The *p* values, least squares mean differences (diff) with 95% confidence intervals and R^2^ values (abundance); *p* values and percent prevalence per study group (prevalence) are reported for the KOs that are more abundant or prevalent amongst infants with AHCGT. Significance was found both overall and during the 31–36 completed weeks postmenstrual age time window. Abbreviations: ns = nonsignificant.

From 24 to 30 weeks PMA (before SHCGT), few statistically significant differences in fecal microbial taxa were observed between study groups, and these taxa were relatively more abundant in the gut microbiome of infants with SHCGT. The relative abundance of *Firmicutes* was significantly higher (*p*= .009, least squares mean difference = 3.24 [0.84, 5.65], R^2^ = 0.10) in infants with any SHCGT versus AHCGT (Figure 2(a)). No specific subtaxon from within *Firmicutes* could account for the observed significant difference. *Actinobacteriota* was also significantly differentially abundant between study groups (*p*= .02, R^2^ = 0.15) from 24 to 30 weeks PMA, mainly attributed to its relatively high abundance in the mildly SHCGT group (least squares mean difference = 2.90 [0.62, 5.18]) (Figure 2(b)), specifically the abundances of *Mycobacteriaceae* (*p*= .0009, least squares mean difference = 1.92 [0.22, 3.63], R^2^ = 0.20) and its genus *Corynebacterium* (*p*= .001, least squares mean difference = 1.76 [0.04, 3.48], R^2^ = 0.19).

From 31 to 36 weeks PMA (during SHCGT), several fecal microbial taxa were significantly less abundant or prevalent in infants with any SHCGT compared to AHCGT. The bacterial phylum *Bacteroidota* was found to be significantly differentially abundant (*p*= .0009, least squares mean difference = −2.93 [−4.59, −1.28], R^2^ = 0.11) between infants with any SHCGT and AHCGT (Figure 2(c)), in addition to its family *Bacteroidaceae* (*p*= .002, least squares mean difference = −2.48 [−3.99, −0.96], R^2^ = 0.087) and its genus *Bacteroides_B* (*p*= .009, least squares mean difference = −1.78 [−3.11, −0.45], R^2^ = 0.087). The bacterial family *Lachnospiraceae* was also found to be both significantly differentially abundant (*p*= .004, least squares mean difference = −2.90 [−4.83, −0.97], R^2^ = 0.095) and prevalent (*p*= .009) between infants with any SHCGT and AHCGT (Figure 2(d)). No singular genera from within *Lachnospiraceae* could account for the observed significant differences. The prevalence of the bacterial family *Ruminococcaceae* was significantly different between study groups (*p*= .007) during this timeframe, which could be attributed to the species *Faecalibacterium prausnitzii* that was significantly less prevalent (*p*= .004) in infants with any SHCGT versus AHCGT (8% vs. 48%). Finally, two families from the bacterial phylum *Firmicutes_C* were additionally significantly less prevalent in infants with any SHCGT versus AHCGT: *Megasphaeraceae* (*p*= .004; 0% vs. 25%) and *Negativicoccaceae* (*p*= .009; 0% vs. 21%). Main genera present in the dataset from these bacterial families included *Megasphaera* and *Anaeroglobus*, and *Negativicoccus*, respectively.

To examine differences in function associated with these taxonomic differences, predictive metagenomic profiling was performed by Tax4Fun2.^36^ Significant KOs (*p*< .05; FP<1%) were tallied by their KEGG pathway classifications to identify where the most changes in function were occurring. Out of the major categories, the greatest number of significant differences in both KO abundance and prevalence occurred in “Metabolism,” and the majority of significant changes in metabolism were related to “Carbohydrate metabolism” (Table 1). The highest number of these KOs significantly depleted in the gut microbiome of infants with any SHCGT compared to AHCGT belonged to the pathway “Starch and sucrose metabolism,” and the second highest number belonged to the pathway “Amino sugar and nucleotide sugar metabolism” (Table 1). All KOs that were significantly less abundant or prevalent were only noted from 31 to 36 weeks PMA (Table 1). The changes in starch and sucrose metabolism could be attributed to the loss of representatives from the phylum *Bacteroidota*, whereas most changes in amino sugar and nucleotide sugar metabolism could be related to the loss of *Lachnospiraceae* (with some overlap of *Bacteroidota*), from referencing each KO (Table 1) with the linked taxonomy in the KEGG database.

### Fecal microbiome features are better predictors of infant head circumference growth trajectories than clinical factors as determined through random forest modeling

**Figure 3.**
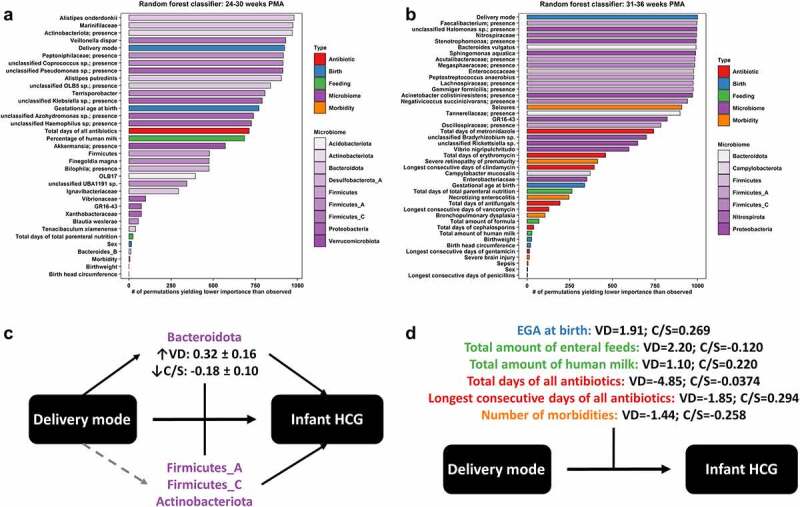
Influence of clinical factors on fecal microbiome and infant head circumference growth relationships. Random forest classifiers were built for predicting appropriate head circumference growth (HCG) trajectory versus any suboptimal HCG trajectory for infants as defined in legend for Figure 1, at the distinct key time points of 24–30 completed weeks postmenstrual age (PMA) (a) and 31–36 completed weeks PMA (b). The relative importance of features was ranked by permutation importance, or the number of permutations yielding lower importance than observed out of 1001. Fecal microbiome features (purple, with shading by bacterial phylum) out ranked most clinical factors, including antibiotics (red), birth (i.e., patient demographic) factors (blue), enteral feeding (green), and morbidity (orange). The exception to this rule was delivery mode, which was examined further by moderation analysis (c + d). Vaginal delivery (VD) significantly (solid black) increased the abundance of fecal *Bacteroidota* (least squares mean and standard error indicated), and the abundance of fecal *Bacteroidota* was both significantly directly associated with infant HCG trajectories and significantly moderated the effect of delivery mode on infant HCG trajectories (c). The abundances of other fecal microbial taxa (see Table 3) were also both significantly directly associated with infant HCG trajectories and significantly moderated the effect of delivery mode on infant HCG trajectories but were not significantly (dashed gray) increased in abundance by VD. Several clinical factors significantly moderated the effect of delivery mode on infant HCG trajectories (d); these clinical factors impacted more specifically VD infants and not Cesarean-section (C/S) delivered infants as indicated by the large differences in Cohen’s D effect sizes (appropriate/any suboptimal HCG trajectory) by delivery mode. That would explain why a significant direct effect of these clinical factors on infant HCG trajectories was mostly not observed

Clinical factors could potentially confound the relationships between the infant fecal microbiome and HCG trajectories, and thus required investigation to probe the robustness of this study’s findings. Both statistical testing and machine learning modeling were used to determine the independent effect of a given clinical factor on infant HCG trajectories, and the relative importance of clinical factors compared to fecal microbiome features in predicting infant HCG trajectories, respectively. Separate random forest classifiers were built for the key time windows of 24–30 weeks PMA (Figure 3(a)) and 31–36 weeks PMA (Figure 3(b)), and in each case fecal microbiome features out ranked the majority of clinical factors in importance for binary classification of infants into AHCGT versus any SHCGT with 77.5% and 84% accuracy, respectively. From 24 to 30 weeks PMA, the top patient demographic factor was gestational age at birth (ranked #13), antibiotic factor was total days of all antibiotics (ranked #16), and enteral feeding factor was percentage of enteral feeds as human milk (ranked #17), which were all in the bottom 2/3rds ranked section of important features (Figure 3(a)). From 31 to 36 weeks PMA, the top patient demographic factor was again gestational age at birth (ranked #29), antibiotic factors were total days of metronidazole (ranked #20), total days of erythromycin (ranked #24) and consecutive days of clindamycin (ranked #26), and enteral feeding factor was total days of total parenteral nutrition (ranked #30), which were all in the bottom 2/3rds ranked section of important features (Figure 3(b)). Further, no statistically significant differences in patient demographics (Table 2), or clinical care including antibiotics and enteral feeding were found between study groups (Table S3). Critically, these results indicate that changes in infant HCG trajectories were not due to caloric restriction.

**Table 2.** Clinical characteristics of the MIND infant cohort at the University of Chicago Comer Children’s Hospital

Demographics/Outcomes	AHCGT	Mildly SHCGT	Moderately SHCGT	Severely SHCGT	*p* value
Mode of delivery, vaginal	42.9% (12)	12.5% (2)	12.5% (1)	16.7% (1)	0.1
Gestational age at birth, completed weeks	28.3 ± 2.6	27.1 ± 2.2	27.0 ± 3.4	26.2 ± 3.1	0.3
Sex, male	46.4% (13)	56.3% (9)	25.0% (2)	66.7% (4)	0.4
Birth weight, kg	1.02 ± 0.38	0.98 ± 0.33	1.07 ± 0.55	0.96 ± 0.53	0.9
Birth head circumference, cm	24.9 ± 2.9	24.7 ± 2.8	24.6 ± 4.1	24.3 ± 3.8	0.9
**Outcomes**	**AHCGT**	**Mildly SHCGT**	**Moderately SHCGT**	**Severely SHCGT**	***p* value**
Bronchopulmonary dysplasia	53.6% (15)	56.3% (9)	62.5% (5)	83.3% (5)	0.6
Necrotizing enterocolitis	0.0% (0)	0.0% (0)	0.0% (0)	50.0% (3)	0.0006
Severe brain injury	7.1% (2)	0.0% (0)	12.5% (1)	50.0% (3)	0.01
Seizures	7.1% (2)	12.5% (2)	12.5% (1)	16.7% (1)	0.7
Sepsis	0.0% (0)	0.0% (0)	25.0% (2)	0.0% (0)	0.03
Severe retinopathy of prematurity	3.6% (1)	12.5% (2)	25.0% (2)	16.7% (1)	0.2
Any listed morbidity	60.7% (17)	56.3% (9)	62.5% (5)	83.3% (5)	0.8
2+ listed morbidities	10.7% (3)	18.8% (3)	25.0% (2)	66.7% (4)	0.03
Length of NICU stay, days	77.9 ± 34.8	83.7 ± 40.6	126.8 ± 90.3	124.2 ± 52.3	0.2
PMA at discharge, completed weeks	39.0 ± 3.8	38.5 ± 4.3	44.5 ± 10.0	43.5 ± 5.0	0.1

Legend: Head circumference growth groups are defined in legend for Figure 1. Binary variables are reported as the percentage of patients (number of patients) per group, with *p* values calculated by the Fisher’s exact test. Numerical variables are reported as the mean ± standard deviation per group, with *p* values calculated by Welch’s ANOVA. Abbreviations: NICU = Neonatal intensive care unit; PMA = Postmenstrual age.

**Table 3.** Infant head circumference growth-associated fecal microbial taxa that significantly moderated vaginal delivery effects

Fecal microbial taxon	Coefficient	95% confidence intervals	*p* value	McFadden’s R^2^
Acutalibacteraceae	1.66	[1.65, 1.67]	<2x10^−16^	0.03
Bacteroidaceae	0.21	[0.20, 0.22]	<2x10^−16^	0.02
*Bacteroides_B*	1.37	[1.35, 1.38]	<2x10^−16^	0.03
*dorei*	0.10	[0.09, 0.11]	<2x10^−16^	0.02
*vulgatus*	1.11	[1.10, 1.12]	<2x10^−16^	0.02
Dialisteraceae	ns			
*Dialister*	ns			
*invisus*	1.06	[1.05, 1.07]	<2x10^−16^	0.02
Erysipelatoclostridiaceae	ns			
*Coprobacillus*	−1.32	[−1.33, −1.31]	<2x10^−16^	0.02
*Erysipelatoclostridium*	1.50	[1.49, 1.51]	<2x10^−16^	0.03
Lachnospiraceae	1.47	[1.44, 1.49]	<2x10^−16^	0.03
*Eubacterium_E*	1.10	[1.09, 1.11]	<2x10^−16^	0.02
*Hungatella*	ns			
*effluvii*	0.88	[0.86, 0.89]	<2x10^−16^	0.03
Megasphaeraceae	ns			
*Megasphaera*	0.97	[0.96, 0.98]	<2x10^−16^	0.03
Mycobacteriaceae	0.82	[0.81, 0.82]	<2x10^−16^	0.02
Rikenellaceae	ns			
*Alistipes*	ns			
*putredinis*	0.21	[0.20, 0.22]	<2x10^−16^	0.02
Ruminococcaceae	ns			
*Faecalibacterium*	1.54	[1.54, 1.55]	<2x10^−16^	0.03
*prausnitzii*	3.70	[3.69, 3.72]	<2x10^−16^	0.03

Legend: Significance of moderation was evaluated by the Wald statistic (*p*< 0.05; FP<1%), model coefficients with 95% confidence intervals and McFadden’s R^2^ on cumulative link mixed regression of infant head circumference growth trajectories for the interaction of delivery mode and abundance of a given fecal microbial taxon, with postmenstrual age, and delivery mode and the given fecal microbial taxon abundance individually as fixed effects, plus patient as a random effect. Head circumference growth groups are defined in legend for Figure 1. Abbreviations: ns = nonsignificant.

The random forest classifiers identified different subtaxa from within the found key microbial taxa for infant HCG as important predictors across the two time windows. From 24 to 30 weeks PMA, the top microbial taxa were the abundances of proteolytic *Bacteroidota* (*Alistipes onderdonkii, Alistipes putredinis* and family *Marinifilaceae* comprised of *Odoribacter* spp. and *Butyricimonas* spp.), with remaining important subtaxa including the abundance of *Bacteroides_B* (phylum *Bacteroidota*), the presence of an unclassified *Coprococcus* species and abundance of *Blautia wexlerae* (family *Lachnospiraceae*), the presence of *Actinobacteriota* and the abundance of *Firmicutes* (Figure 3(a)). Other unique microbial taxa identified amongst the top third of important features were the abundances of *Veillonella dispar* and an unclassified *Klebsiella* species, and the presence of the bacterial family *Peptoniphilaceae* (Figure 3(a)). From 31 to 36 weeks PMA, the top microbial taxon was the presence of the genus *Faecalibacterium*, with remaining important sub-taxa including the abundance of *Bacteroides vulgatus* and presence of family *Tannerellaceae* (phylum *Bacteroidota*), the presence of families *Lachnospiraceae* and *Megasphaeraceae*, and the presence of *Negativicoccus succinicivorans* (family *Negativicoccaceae*) (Figure 3(b)). Other unique microbial taxa identified amongst the top third of important features were the abundances of bacterial family *Enterococcaceae* and *Peptostreptococcus anaerobius*, and the presences of bacterial family *Acutalibacteraceae* and *Gemmiger formicilis* (Figure 3(b)).

However, it was found that infants with any SHCGT experienced a significantly greater incidence of specific morbidities in the NICU (Table 2). These results contrasted the random forest classifiers, as the model built from 24 to 30 weeks PMA had ranked the incidence of any morbidity amongst the bottom three predictors (Figure 3(a)), and the model built from 31 to 36 weeks PMA had its highest ranked morbidities amongst the bottom 2/3rds ranked section of important features including incidence of seizures (ranked #16) and severe retinopathy of prematurity (ranked #25) (Figure 3(b)). Part of the discrepancy may lie with the incidence of morbidities being correlated with the structure of the infant microbiome, as prior studies have associated specific morbidities, such as necrotizing enterocolitis and sepsis, with the infant fecal microbiota.^42^ Evaluating differences between the infant fecal microbiome amongst patients with and without specific morbidities is beyond the scope of this study; it was instead important to ensure that morbidity did not significantly alter our found relationships between infant fecal microbial taxa and HCG trajectories. A robust permutation approach was utilized to select and rank features for the random forest classifiers,^43^ which more frequently identified microbiome characteristics over morbidities as important for predicting infant HCG trajectories. As an additional precaution, a limited morbidity (LM) subset was created to determine the dependence of the fecal microbial taxon abundance associations with HCG trajectories on this confounder. This LM subset comprised of infants without severe brain injury, severe retinopathy of prematurity, necrotizing enterocolitis, seizures, or sepsis. Not only were the previously established gut microbiome and infant HCG trajectory relationships retained after consideration of the clinical variables, but *Actinobacteriota* abundance newly emerged as a potential biomarker distinguishing moderately to severely SHCGT from mildly SHCGT and AHCGT (Figure 2(b)).

Redundancy analysis confirmed the significant difference (*p*= .001) in gut microbiome β-diversity between study groups of the LM subset for both the model and terms, including PMA (R^2^ = 0.6829) and any SHCGT (R^2^ = 0.3706). For infants with any SHCGT versus AHCGT, significant reductions of *Bacteroidota* (Figure 2(c)) and *Lachnospiraceae* (Figure 2(d)) abundance were also confirmed in the LM subset overall (*p*= .008, least squares mean difference = −0.53 [−0.92, −0.15], R^2^ = 0.086 and *p*= .005, least squares mean difference = −0.51 [−0.86, −0.17], R^2^ = 0.085 respectively). No sub-taxon from within *Bacteroidota* and *Lachnospiraceae* was significantly differentially abundant between study groups (Table S4). Furthermore, predicted metagenomic profiling again revealed that the greatest number of changes in function occurred in “Metabolism,” specifically “Carbohydrate metabolism” and its subcategories “Starch and sucrose metabolism” and “Amino sugar and nucleotide sugar metabolism” (Table S5).

A finding unique to the LM subset as compared to the complete dataset was that infants with moderately to severely SHCGT had a significant depletion in overall *Actinobacteriota* abundance (*p*= .008, least squares mean difference = −0.78 [−1.34, −0.21], R^2^ = 0.086) compared to infants with AHCGT or mildly SHCGT (Figure 2(b)). Again, no specific sub-taxon could account for this significant difference (Table S4). Interestingly, the mean abundances of *Bacteroidota* (Figure 2(c)) and *Lachnospiraceae* (Figure 2(d)) also more closely followed the found trends between infant HCG and the gut microbiome, particularly for infants with severely SHCGT, in the LM subset versus the complete dataset. It is not expected that an “immature” microbiome would be the sole effector of SHCGT, and as infants with severely SHCGT experienced the highest incidence of morbidities (Table 2), this study group was most impacted by the confounder. When examining the mean abundances of *Bacteroidota* (Figure 2(c)) and *Lachnospiraceae* (Figure 2(d)) between infants with mildly versus moderately to severely SHCGT in the LM subset, however, no significant differences were observed. Taken together, after consideration of the confounding variables, the increase in severity of SHCGT is apparently resultant from a continual depletion in key microbial taxa, with first a reduction in the abundances of *Bacteroidota* and *Lachnospiraceae* leading to mildly SHCGT and then a further reduction in the abundance of *Actinobacteriota* leading to moderately to severely SHCGT.

Clinical characteristics were statistically analogous between the complete dataset and the LM subset (Table S6). However, a lower proportion of infants with any SHCGT in the complete dataset were vaginally delivered (Table 2), and this difference became statistically significant (*p*= .02) in the LM subset (Table S6). Interestingly, vaginal delivery was also identified as a highly important predictor for the random forest classifiers across both time windows, ranking #5 from 24 to 30 weeks PMA (Figure 3(a)) and as the top feature from 31 to 36 weeks PMA (Figure 3(b)). We hypothesized that the positive influence of vaginal delivery on infant HCG trajectories resulted from the vertical transmission of beneficial microbes,^44^ and thus investigated the interaction of this clinical factor with the infant microbiome in further detail.

### The impact of delivery mode on infant head circumference growth trajectories reveals dispersal limitation and supersedes habitat filtering as a driver of optimal infant microbiome succession

Vaginal delivery was significantly positively associated with infant HCG trajectories (Table S6; Figure 3(a,b)), and we hypothesized that this resulted from the vertical transmission^44^ of microbes that elicit AHCGT. To address this hypothesis, it was first evaluated if vaginal delivery’s benefit to infant HCG trajectories was affected by the structure of the microbiome through moderation analysis. Significant moderation by the abundances of fecal microbial taxa previously significantly associated with AHCGT (Figures 2(c,d) and 3(a,b)) was indeed found, including *Lachnospiraceae, Bacteroides_B, Faecalibacterium* and *Megasphaera* (Table 3). Next, it was investigated if the abundances of fecal microbial taxa were significantly associated with delivery mode to determine which microbes were more likely to be vertically transmitted. It was discovered that the abundance of *Bacteroidota* was significantly enhanced by vaginal delivery (Figure 3(c)), which was one of the found key microbial taxa for infant HCG (Figure 2(c)). Taken together, these results affirm our hypothesis that delivery mode can be considered as a dispersal limitation factor influencing optimal infant microbiome succession, which impacts infant health and development. Understanding the relative importance of successional drivers is vital for identifying potential indirect routes of modifying the gut microbiome-brain axis, or variables that could alter the effectiveness of direct interventions. Despite clinical variables typically attributed as habitat filtering factors impacting microbiome succession, such as enteral feeding and antibiotics, not having a significant direct effect on infant HCG trajectories (Table 2), it was interrogated if these clinical factors could significantly moderate the influence of vaginal delivery. That was indeed found to be the case (Figure 3(d)), therefore delineating that optimal infant microbiome succession is primarily driven by dispersal limitation and secondarily by habitat filtering, at least in the context of host neurodevelopmental markers.

To test the influence of the microbiome on the relationship between delivery mode and infant HCG trajectories, moderation analysis was conducted. Significant moderation (*p*< .05; FP<1%) by fecal microbial taxa that were consistently augmented or depleted in abundance for vaginally delivered infants with AHCGT versus all three SHCGT categories are displayed in Table 3. Vaginally delivered infants with any SHCGT compared to vaginally delivered infants with AHCGT had reductions in fecal microbial taxa previously found to be directly significantly associated with infant HCG trajectories from both standard statistics (Figure 2(c,d)) and machine learning modeling (Figure 3(a,b)), including bacterial family *Bacteroidaceae* and its genus *Bacteroides_B* (also its species *Bacteroides dorei* and *Bacteroides vulgatus*), bacterial family *Lachnospiraceae* (also its genus *Eubacterium_E* and *Hungatella effluvii), Faecalibacterium prausnitzii* and bacterial genus *Megasphaera* (from family *Megasphaeraceae*) (Table 3). Additional diminished fecal microbial taxa that were selected as predictors of infant HCG trajectories for the random forest classifiers only (Figure 3(a,b)) included the bacterial family *Acutalibacteraceae* and *Alistipes putredinis* (Table 3). Fecal microbial taxa that were decreased in abundance in vaginally delivered infants specifically across all SHCGT categories compared to AHCGT included *Dialister invisus*, the bacterial genus *Erysipelatoclostridium*, and the bacterial family *Mycobacteriaceae*, whereas the bacterial genus *Coprobacillus* was the only fecal microbial taxon found to be increased specifically in vaginally delivered infants across all SHCGT categories versus AHCGT (Table 3). The large number of significant fecal bacterial modifiers, with many of these having a significant direct impact on infant HCG trajectories, indicate that the positive effect of vaginal delivery on infant HCG trajectories is likely dependent on the microbiome (Figure 3(c)).

To statistically test the hypothesis of vertical transmission, the differences in fecal microbial taxon abundances between vaginally delivered versus Cesarean-section delivered infants were assessed by ANOVA on multivariate regression with PMA as a fixed effect and patient as a random effect. *Bacteroidota* (*p*= .01, least squares mean difference = 0.50 [0.12, 0.88], R^2^ = 0.08), a microbial taxon directly significantly associated with infant HCG trajectories by both standard statistics (Figure 2(c)) and machine learning modeling (Figure 3(a,b)), was found to be significantly increased in abundance in vaginally delivered infants compared to Cesarean-section delivered infants. This result also included its genus *Bacteroides_B* (*p*= .009, least squares mean difference = 0.52 [0.14, 0.90], R^2^ = 0.08), as well as its family *Tannerellaceae* (*p*= .008, least squares mean difference = 0.47 [0.13, 0.82], R^2^ = 0.04), genus *Parabacteroides* (*p*= .007, least squares mean difference = 0.49 [0.14, 0.84], R^2^ = 0.05) and species *Parabacteroides distasonis* (*p*= .009, least squares mean difference = 0.55 [0.14, 0.95], R^2^ = 0.06). An additional fecal microbial taxon significantly elevated in abundance in vaginally versus Cesarean-section delivered infants but not found to be directly related to infant HCG trajectories was the bacterial family *Methanobacteriaceae* (*p*= .005, least squares mean difference = 0.41 [0.12, 0.69], R^2^ = 0.03) and its genus *Methanobrevibacter_A* (*p*= .009, least squares mean difference = 0.38 [0.09, 0.66], R^2^ = 0.03). Therefore, *Bacteroidota* is likely vertically transmitted in vaginally delivered infants, which can be considered as a mediator of the positive influence of vaginal delivery on infant HCG trajectories due to both its significant direct effects on infant HCG trajectories and its significance as a moderator of the relationship between delivery mode and infant HCG trajectories (Figure 3(c)). Other fecal microbial taxa that colonize the infant fecal microbiome potentially less specifically via vertical transmission additionally served as moderators, as previously described.

Finally, to test how clinical factors change infant HCG trajectory outcomes of vaginally delivered versus Cesarean-section delivered infants, moderation analysis equivalent to the approach used for the fecal microbial taxon abundances was performed. Time was found to be an important moderator of delivery mode and infant HCG trajectory relationships as demonstrated by the significance of gestational age at birth (*p*< 2x10^−16^, coefficient = 13.035 [13.030, 13.039], R^2^ = 0.11), and its proxies’ birthweight (*p*< 2x10^−16^, coefficient = 16.220 [16.216, 16.224], R^2^ = 0.11) and birth head circumference (*p*= .002, coefficient = 9.697 [3.669, 15.725], R^2^ = 0.08). Vaginally delivered infants with SHCGT were all born <27 weeks gestational age, whereas only 17% of vaginally delivered infants with AHCGT were born <27 weeks gestational age, and this trend can clearly be observed for the likely vertically transmitted *Bacteroidota* (Figure 2(c)), as well as *Lachnospiraceae* (Figure 2(d)), which appear relatively high in abundance for infants with SHCGT <27 weeks PMA with a subsequent rapid decline. Instead, skin bacteria from the phyla Firmicutes (Figure 2(a)) and *Actinobacteriota* (particularly *Corynebacterium* spp. as previously determined to be significantly augmented in infants with mildly SHCGT) (Figure 2(b)) were relatively and more stably higher in abundance for these earlier time points for infants with SHCGT. In contrast, infants with AHCGT from 27 weeks PMA onward had relatively higher and stable abundances of *Bacteroidota* (Figure 2(c)) and *Lachnospiraceae* (Figure 2(d)) and lower earlier abundances of *Firmicutes* (Figure 2(a)) and *Actinobacteriota* (Figure 2(b)). The number of morbidities was expectedly also a significant modifier (*p*= 3x10^−11^, coefficient = −6.090 [−8.649, −3.530], R^2^ = 0.13), but sex was not (*p*= .7, coefficient = 0.534 [−2.409, 3.476], R^2^ = 0.02). Both time (Figure 1(c)) and morbidities (Table 2) were found to have a significant direct effect on infant HCG trajectories, which is why the previous interrogation of critical time points (change point analysis) and morbidity as a confounder (LM subset) was undertaken. Intriguingly, several clinical factors known to affect the infant microbiome were found to be significant modifiers of the effect of delivery mode on infant HCG trajectories, and these had at least a fivefold greater impact on vaginally delivered infants, whose Cohen’s D effect sizes (AHCGT/any SHCGT) were all large, compared to Cesarean-section delivered infants, whose Cohen’s D effect sizes were all small to negligible (Figure 3(d)). These clinical factors included both the total days (*p*= 2×10^−5^, coefficient = −44.417 [−64.711, −24.124], R^2^ = 0.18) and longest number of consecutive days (*p*= 4×10^−8^, coefficient = −18.518 [−25.100, −11.935], R^2^ = 0.13) of all antibiotics, and the total amount of enteral feeds (*p*< 2×10^−16^, coefficient = 12.594 [12.590, 12.597], R^2^ = 0.14), along with the additional proxies of total days of total parenteral nutrition (*p*= 2×10^−6^, coefficient = −8.913 [−12.546, −5.279], R^2^ = 0.14) and the day of life full enteral feeding was achieved (*p*= .002, coefficient = −12.888 [−21.230, −4.546], R^2^ = 0.12). As for enteral feeding-type, the total amount of human milk was a significant moderator (*p*< 2×10^−16^, coefficient = 8.292 [8.288, 8.297], R^2^ = 0.07), whereas the total amount of formula was not (*p*= .1, coefficient = 2.603 [−0.738, 5.943], R^2^ = 0.04). Notably, none of these clinical factors had a significant direct effect on infant HCG trajectories (Table 2). As diet and antibiotics are known to be primary influencers of the intestinal environment, these results demonstrate that delivery mode, i.e., a dispersal limitation factor from vertical transmission, supersedes habitat filtering as a driver of infant gut microbiome succession.

## Discussion

There is a vital need to identify modifiable environmental factors for reducing the incidence of developmental impairments at a time point early enough for successful intervention. Thus, it was the aim of this study to determine if infant gut microbiome composition was associated with HCG, the earliest marker of neurodevelopment.^13,14^ This study is the first to show that β-diversity of the gut microbiome was significantly distinct between infants with AHCGT versus any SHCGT (Figure 1(a)), and that reduced abundances of *Bacteroidota* (Figure 2(c)) and *Lachnospiraceae* (Figure 2(d)) were specifically associated with SHCGT independent of concurrent morbidities and caloric restriction. Notably, this study’s novel application of change point analysis further linked the microbiome as a potential mediator of HCG by revealing that the timing of peak gut microbiome composition alteration (Figure 1(b)) exactly matched the timing of significant HCG separation between study groups (Figure 1(c)) at 30 weeks PMA. We hypothesized that clinical variables cause deviations in head circumference growth by altering the gut microbiome; in other words, the gut microbiome is a mediator of infant HCG. The clinical variable would thus occur first, and then as the gut microbiome is impacted, head circumference growth ceases, i.e., changes to the infant gut microbiome and head circumference growth would occur concurrently as observed. The preceding casual clinical factors were therefore additionally thoroughly examined to determine their influence on gut microbiome successional patterns that result in infant HCG trajectory deviations, of which the significant effect of delivery mode (Table S6; Figure 3(a); Figure 3(b)) provided further innovative insights into the primary drivers of optimal infant microbiome maturation.

There exists evidence for the potential mechanistic impact of *Bacteroidota* and *Lachnospiraceae* on host neurodevelopment. *Bacteroidota* may affect neurodevelopment through altering intestinal barrier integrity and systemic metabolite availability. Hsiao and colleagues^45^ have demonstrated that oral treatment of the maternal immune activation mouse model of ASD with human-derived *Bacteroides fragilis* both altered intestinal tight junction protein expression and the serum metabolite profile, and ameliorated behavioral defects. *Lachnospiraceae* are key producers of short-chain fatty acids after fermentation, particularly butyrate,^46,47^ that are utilized by intestinal epithelial cells as a fuel source,^48^ have potent anti-inflammatory effects,^49^ and promote immune system maturation by increasing the colonic population of T regulatory cells.^50–53^ Stolp and colleagues^54^ have shown that neonatal systemic inflammation in rats can lead to altered blood-brain barrier permeability and behavior, and therefore, *Lachnospiraceae* could affect neurodevelopment through influencing energy resources and immunity.

Vaginal delivery was the clinical factor significantly associated with improved HCG (Table S6; Figure 3(a,b)) but was dependent upon the successful vertical transmission^44^ of key microbial taxa (Figure 3(c); Table 3), which could be impeded by certain clinical factors, including prolonged antibiotics, delayed enteral feeding, formula feeding and younger gestational age at birth leading to SHCGT even with vaginal delivery (Figure 3(d)). The significance of gestational age connects to the change point analysis demonstrating 30 weeks PMA to be a key transition point for the preterm infant gut microbiome (Figure 1(b)), which complements other studies.^21,24^ This transition is thought to occur due to intestinal maturation,^55^ at which point the preterm infant intestinal environment is like the term infant at birth. Studies in term infants using different neurodevelopmental metrics and timelines have also indicated *Bacteroidota* and *Lachnospiraceae* as potentially important taxa; Kelsey et al.^56^ found functional brain network connectivity was associated with *Bacteroides* and *Lachnospiraceae* abundance, and Loughman and colleagues^57^ associated behavioral problems at 24 months of age with a low *Prevotella* abundance at 12 months. Therefore, the results from this study taken in the context of the current literature interestingly suggest that *Bacteroidota* and *Lachnospiraceae* should broadly colonize infants born >30 weeks gestational age at birth through vaginal delivery.

Diet and antibiotics are known to have a large impact on the infant microbiome, exceeding stochasticity.^30,44,58^ Further, a seminal study by Feng and colleagues has demonstrated that the influence of relative microbial fitness on infant microbiome succession appears to supersede historical contingency, i.e., the order of introduction of microbes.^59^ These results have together indicated that habitat filtering, i.e., variables that shape the intestinal environment, is a dominant driver of infant microbiome succession. Other studies have additionally shown that delivery mode shapes the initial colonizers of the infant gastrointestinal tract;^44,58^ these initial colonizers are predominantly skin-sourced microbes for Cesarean-section delivered infants, and predominantly fecal-sourced microbes for vaginally delivered infants, especially *Bacteroidota*^60,61^ which is a finding that complements our study. However, infants delivered at term possess an advantage as they can access the home environment usually relatively quickly, whereas preterm infants usually remain in the hospital environment at length with sanitation protocols preventing normal microbial dissemination.^62^ This difference allows the effect of dispersal limitation on infant microbiome succession to be more closely examined in preterm infants, and a major finding of this study is that its influence exceeds habitat filtering. That could explain why studies of the effects of enteral feeding^20,58^ and antibiotics^58,63,64^ on health and microbiome composition have yielded somewhat inconsistent results for preterm infants, despite their known effects on the term infant microbiome.

However, this study’s findings do not preclude the importance of habitat filtering as a driver, since after considering dispersal limitation, significance for antibiotics, caloric restriction, and human milk feeding was found (Figure 3(d)). Human milk provides microbially fermentative human milk oligosaccharides,^65^ which are known to be degraded by *Bacteroidota, Lachnospiraceae*, and *Bifidobacterium*^65,66^ and additionally supported by our predicted functional profiling analysis. Interestingly, this microbial activity can in turn support infant growth through increasing the energy yielded from the dietary substrate and influencing how the host uptakes the available energy. Human milk oligosaccharides are both generally indigestible^67,68^ and minimally absorbed^69^ by the host itself, but the short-chain fatty acids produced from microbial fermentation can be consumed by the host as a caloric source.^48^ Further, short-chain fatty acids can regulate systemic host gene expression, including control of intestinal barrier function^49^ and subsequent energy metabolism.^48^ Notably, human milk can also act as a source of microbes and thus a secondary dispersal limitation factor.^70^ However, it is unclear if human milk feeding can recapitulate the microbial diversity lost from Cesarean-section delivery, as studies aiming to restore the gut microbiome in Cesarean-section delivered infants using both maternal vaginal swabs and human milk feeding have yielded a partial benefit at best.^71–74^ The presented work has indicated the importance to host neurodevelopment of *Bacteroidota* (Figure 2(c)) and *Lachnospiraceae* (Figure 2(d)), which are obligately anaerobic fecal microbes not native to the vaginal^75–77^ or human milk^70^ flora, potentially explaining the observed outcomes from these studies. A proof-of-concept study by Korpela et al. even directly compared fecal microbiota transplantation to vaginal swabs for gut microbiome restoration in infants delivered by Cesarean-section and demonstrated that only fecal microbiota transplantation allowed for full restoration of the gut microbiome in comparison to vaginally delivered infants, with *Bacteroides* particularly indicated in the observed difference between methods.^78^ A precaution for these studies approaches is that the maternal microbiome may not be optimal or could exhibit dysbiosis, as the fecal and vaginal microbiome composition of mothers that deliver preterm has exhibited significant differences compared to mothers that deliver at term in other studies.^77,79^ The effects of delivery mode on preterm infant health outcomes and microbiome succession can thus also be masked depending on a given study’s cohort distribution.^58^ Therefore, future studies on infant gut microbiome remediation could focus on the use of defined microbial mixtures for both enhanced safety and targeted efficacy, as has been done for the treatment of recurrent *Clostridioides difficile* infection in adults.^80^ Further, it is recommended that clinical variables should be considered sequentially for infant microbiome studies, first by dispersal limitation factors (e.g., delivery mode, environment such as hospital versus home or urban versus rural, probiotics) then by habitat filtering factors (e.g., diet, prebiotics, antibiotics).

There are several limitations in our study. First, the completed 16S rRNA gene sequencing technique produces relative abundance data. An appropriate compositional data analysis framework^81^ was utilized to ensure statistical fidelity of abundance differences in specific microbial taxa such as *Bacteroidota* (Figure 2(c)) and *Lachnospiraceae* (Figure 2(d)), but changes to the overall bacterial load cannot be detected by this approach, which can occur after antibiotic treatment for example. Further, the accuracy of predicted metagenomic profiling is dependent upon the coverage of the KEGG database for the microbial taxa present in a given dataset and the consistency of a present functional gene across strains of the same species. A cautious interpretation of this data was employed through rigorous validation against background literature, and since *Bacteroidota, Lachnospiraceae* and *Bifidobacterium* are well described carbohydrate degraders,^65,66^ the main finding from this study of a reduced carbohydrate capacity of the gut microbiome in infants with any SHCGT is not incongruent. Finally, we had lower sample numbers that were consistent with other studies of infant neurodevelopment,^56,82^ particularly for preterm infants that were vaginally delivered. Although precision and the effect sizes that could be significantly detected were impacted by the lower sample numbers, this study was nonetheless able to uncover differences in the gut microbiome between infants with AHCGT versus SHCGT that had effect sizes with respective 95% confidence intervals of high magnitude, suggesting repeatability in future studies under equivalent assumptions.

*Bacteroidota* and *Lachnospiraceae* are both known core taxa of the adult gut microbiome,^83^ and our work provides evidence that *Bacteroidota* and *Lachnospiraceae* need to integrate into the gut microbiota early in infancy for optimal developmental outcomes. Ultimately, the promising findings from this study encourage future research of microbiome modification to improve infant developmental trajectories, either from clinical investigations to enhance statistical rigor and reproducibility or verify microbial load, functions and metabolites, or animal model experiments for direct probing of the impact of *Bacteroidota* and *Lachnospiraceae* on neurodevelopment. Optimizing the gut microbiome in infancy could also reduce the incidence of developmental disability, as evidenced by the associations between early antibiotics and later cognitive outcomes^25,26^ or the altered gut microbiome of children with ASD,^32^ and ADHD,^33^ and future work could continue to follow the fecal microbiome of infants with AHCGT and SHCGT over time to determine the potential lifelong effectiveness of an infant microbiome-based intervention.

## Patients and methods

### Study participants

The Microbiome In Neonatal Development (MIND) study received approval from an institutional review board (IRB16-1431). Study participant enrollment took place in the NICU at the University of Chicago Comer Children’s Hospital between January 2010 and December 2018. Infants born prior to 37 weeks gestational age were eligible for the study and were enrolled after receiving written informed consent from the parent. Infants with a genetic syndrome or severe congenital anomalies, including major congenital heart disease or major kidney, lung, or brain malformation, were excluded, as were infants judged not to be viable by the attending physician.

### Clinical data and sample collection

For assessment of the infant fecal microbiome and HCG, infant diapers were collected, and head circumferences measured weekly by nursing staff. HCG trajectory was evaluated by the difference between head circumference z-scores calculated from the Fenton 2013 growth curve^39^ by the completed weeks method at birth and 36 weeks PMA. If the infant was discharged prior to 36 weeks PMA, the measurement at NICU discharge was taken instead (no infant was discharged prior to 34 weeks PMA). Infants that expired in the NICU were excluded. Clinical variables identified as possible confounders were additionally gathered by abstracting information from patient charts using the electronic medical record system at the University of Chicago Comer Children’s Hospital. Data collected included delivery mode, gestational age at birth, sex, birthweight, head circumference at birth, enteral feeding regimens (mean mL per kg bodyweight over time periods of interest), antibiotics administration (total days or longest number of consecutive days administered over time periods of interest), clinical morbidities, length of stay in the NICU and PMA at NICU discharge. Enteral feeding regimens were described by the amount of human milk, formula or total enteral (human milk + formula), with the days of total parenteral nutrition (0 mL/kg total enteral nutrition) and day of life total enteral nutrition was achieved (120 mL/kg) recorded. Antibiotics were described both as a total and divided into their pharmacological classes. The morbidities reported include bronchopulmonary dysplasia, severe brain injury, severe retinopathy of prematurity, necrotizing enterocolitis, seizures and sepsis. Bronchopulmonary dysplasia was defined as the need for supplemental oxygen at 36 weeks PMA.^84^ Severe brain injury was defined as the presence of periventricular leukomalacia and/or grade 3 or 4 intraventricular hemorrhage on a cranial ultrasonogram.^85^ Severe retinopathy of prematurity was defined as unilateral or bilateral retinopathy of prematurity at stage 4 or 5 and/or retinopathy of prematurity requiring treatment by laser or antivascular endothelial growth factor drugs.^85^ Necrotizing enterocolitis was defined by the modified Bell’s criteria, with at least stage 2 being requisite for disease classification.^86^ Presence of seizures required both clinical and EEG-activity confirmation. Sepsis encompassed both blood culture-positive early- (<72 h) and late- (>72 h) onset.

### Illumina 16S rRNA gene sequencing and processing

Patient fecal samples were submitted to the Environmental Sample Preparation and Sequencing Facility at Argonne National Laboratory (Lemont, IL, USA) for genomic DNA extraction and Illumina 16S rRNA gene sequencing.^34,35^ Data retrieved from the facility were subsequently processed and merged by the sample inference tool DADA2^87^ from within QIIME2 version 2019.7^88^. The processed and merged data was then classified to the genus level by the IDTAXA method^89^ via R package DECIPHER version 2.14.0 using the Genome Taxonomy Database^90^ version 89, and additionally into species-like groups by the online NCBI Nucleotide Basic Local Alignment Search Tool^91^ (BLAST – https://blast.ncbi.nlm.nih.gov/Blast.cgi) with an identity threshold of ≥97%. After classification, low quality samples with <1000 total sequence counts were removed, and then species-like groups that represented <0.1% mean abundance were culled.

The α-diversity metrics of richness and Shannon diversity were computed by R package iNEXT^92^ version 2.0.20. For β-diversity analysis, the taxonomic levels of phylum, family, genus, and species were individually considered. These data were center-log ratio transformed using R package ALDEx2^93^ version 1.18.0. Predicted functional profiles were obtained via the R package Tax4Fun2^36^ version 1.1.5 using their downloaded version 2 reference dataset from NCBI BLAST (Ref99NR) and KEGG.^41^ This data was center-log ratio transformed with zeroes imputed by the nonparametric multiplicative simple method through R package zCompositions version 1.3.4.

### Statistical analysis

All statistical analysis was done in R statistical software version 3.6.2 with plots generated by R package ggplot2 version 3.3.0. Original study groups were defined by the loss in head circumference z-score from birth to 36 weeks PMA: AHCGT (≥0.5; n = 28 patients, n = 118 fecal samples), mildly SHCGT (<0.5–1; n = 16 patients, n = 67 fecal samples), moderately SHCGT (<1-1.5; n = 8 patients, n = 32 fecal samples) and severely SHCGT (<1.5; n = 6 patients, n = 23 fecal samples). Later analysis combined all SHCGT groups in comparison to the AHCGT group, and the moderately to severely SHCGT groups in comparison to the AHCGT to mildly SHCGT groups, for both the complete and LM datasets. The LM subset comprised of infants without severe brain injury, severe retinopathy of prematurity, necrotizing enterocolitis, seizures, or sepsis; bronchopulmonary dysplasia was not excluded as it had an equivalent risk ratio between study groups (Table 2): AHCGT (n = 23 patients, n = 101 fecal samples), mildly SHCGT (n = 13 patients, n = 50 fecal samples) and moderately to severely SHCGT (n = 8 patients, n = 32 fecal samples).

Differences in the clinical characteristics of delivery mode, sex, and incidence of morbidities between study groups were assessed by the Fisher’s exact test and presented as the percentages (number) per group. Differences in the remaining clinical variables between study groups were assessed by Welch’s ANOVA (multiple groups) or Welch’s *t*-test (two groups) and presented as the mean ± standard deviation per group. Post-hoc analysis was conducted by the Games-Howell method using R package userfriendlyscience version 0.7.2, and pairwise Cohen’s Ds were computed by R package rstatix version 0.6.0 without assuming equal variances. Detailed statistics are provided for the complete dataset (Table S3) and for the LM subset (Table S6).

For multidimensional β-diversity analysis, redundancy analysis was performed by R package vegan version 2.5.6 on the genus level data, with HCG trajectory and PMA as environmental variables, and permutations blocked by patient. Details of the redundancy analysis are provided (Table S9).

For determining statistically significant differences in α-diversity metrics, individual microbial taxon abundances or predicted function (KEGG orthologies – KOs) abundances between study groups both overall and separately for the key time windows of 24–30 weeks PMA and 31–36 weeks PMA, mixed-effect linear models were constructed via R package lme4 version 1.1.23 using HCG trajectory and PMA (overall only) as fixed effects, and patient as a random effect. Additionally, an equivalent approach was utilized to test which microbial taxon abundances were resultant from vertical transmission, by replacing the fixed effect of HCG trajectory with delivery mode. Variables were standardized using R package standardize version 0.2.1 prior to fitting the models, and *p* values were determined by Satterthwaite’s method using R package lmerTest version 3.1.2. For the α-diversity metrics, a standard *p* value <.05 was considered significant. To correct for multiple testing of the individual microbial taxa and KOs, the Benjamini-Hochberg method was utilized, and a false-positive rate of <1% was the set threshold for significance. Least squares mean differences (i.e., mean difference after accounting for other fixed and random effects) with their respective 95% confidence intervals are reported (AHCGT vs. SHCGT or vaginal vs. Cesarean-section delivery). The marginal coefficient of determination for generalized mixed-effect models (variance explained by fixed effects) calculated by R package MuMIn version 1.43.15 was also reported as the R^2^.

Prevalence of individual microbial taxa or KOs amongst patients within study groups was also computed overall and separately for the key time windows of 24–30 weeks PMA and 31–36 weeks PMA. If a given microbial taxa or KO had >0 sequence counts in at least one fecal sample during the time window of interest, it was considered to be present in the patient. Statistical significance was then evaluated between study groups by the Fisher’s exact test, with the Benjamini-Hochberg correction for multiple testing applied. A false-positive rate of <1% was the set threshold for significance. The ratios of study group percent prevalence are also reported.

Discussed key microbial taxa for HCG were both significantly differentially abundant or prevalent between study groups by the set threshold and were present in at least three patients of one study group. For KOs, statistically significant features were tallied by their respective KEGG^41^ pathways using R package KEGGREST version 1.26.1. The importance of KEGG pathways for discussion was then determined through ranking them by their total number of significant features. Detailed statistics from all studied datasets and time windows are provided for both the abundance and prevalence of significant microbial taxa and KOs (Table S10). This same procedure was applied to discussed vertically transmitted microbial taxa that were significantly differentially abundant by delivery mode; detailed statistics are provided (Table S7).

### Change point analysis

Individual microbial taxa were selected for change point analysis if they significantly varied in abundance by PMA as determined from the above mixed-effect linear models. Change point analysis was conducted on the mean percent abundances of the microbial taxon over PMA, and separately for each study group. Only the patients for which the microbial taxon was present in at least one fecal sample (>0 sequence counts) were used for determining change points of that microbial taxon. A data point for each PMA week from 24 to 36 was required for this analysis, and missing points were imputed by R package imputeTS version 3.0. Change points were evaluated using the pruned exact linear time (PELT) algorithm with a nonparametric cost function based on the empirical distribution of the data using R package changepoint.np^94^ version 1.01. The ideal penalty value (and thus number of change points) was selected from the diagnostic plot as outlined by Lavielle.^95^ The change points of all individual microbial taxa were then tallied by PMA week for each examined taxonomic level (phylum, family, genus, and species), which revealed the time point at which the most changes in microbial taxon abundances were occurring.

### Random forest classification

The R package caret version 6.0.86 was utilized to build random forest classifiers with 500 trees, and the number of variables randomly sampled as candidates at each split (argument *mtry*) tuned from a random search using 10-fold adaptive cross-validation and 3 repeats. For evaluating feature importance, statistical significance was estimated for the decrease in Gini coefficient from permuting the response variable by R package pRF version 1.2. Features were then ranked by their permutation importance,^43^ or the number of permutations yielding a lower importance than observed out of 1001. The key time windows of 24–30 weeks PMA and 31–36 weeks PMA were each separately considered for building random forest classifiers to predict the binary outcome of AHCGT versus any SHCGT.

Classifiers were first built using the fecal microbial taxonomic median abundance and prevalence data individually at the studied taxonomic levels. Microbial taxa that had a statistically significant (*p*< .05) feature importance, were present in at least three patients’ fecal samples and were classified to the respective taxonomic level examined were subsequently selected to be included in the final multi-taxonomic level random forest classifiers. Clinical data were added to the multi-taxonomic level random forest classifiers, which included mode of delivery, gestational age at birth, sex, birthweight, birth head circumference, morbidities, enteral feeding, and antibiotics. After the multi-taxonomic level random forest classifiers were built, a further feature reduction step took place to remove redundant variables through selecting the best descriptors for each feature after ranking by importance. The final random forest classifiers accuracies are described by the subtraction of the out of bag error from 100, and features are displayed by their permutation importance. Details of the random forest classifiers are provided (Table S11).

### Moderation analysis

Cumulative link mixed regression models were built using R package ordinal version 2019.12.10, with infant HCG trajectory (AHCGT -> Mildly SHCGT -> Moderately SHCGT -> Severely SHCGT) as the outcome, PMA, delivery mode, the microbial taxon abundance/clinical factor and the interaction between delivery mode and the microbial taxon abundance/clinical factor as fixed effects, and patient as a random effect. Data were standardized using R package standardize version 0.2.2 prior to fitting the models. Significance of the delivery mode and microbial taxon abundance/clinical factor interaction was assessed by the Wald statistic implemented in the ordinal package. The model coefficients of the interaction between delivery mode and the microbial taxon abundance/clinical factor (i.e., expected change on the log odds scale as HCG severity decreases) with their respective 95% confidence intervals are provided. McFadden’s R^2^ was calculated manually through dividing the log likelihood of the model by the log likelihood of the null model and subtracting this value from one.

The Benjamini-Hochberg method was used to correct for multiple testing, and a false-positive rate of <1% plus a value of >|0.1| for the model coefficient was the set threshold for significance. Additionally, only the microbial taxa that met the prevalence threshold for assessing the effects of delivery mode on microbial taxon abundances as described in the statistical analysis section were considered. As it was of specific interest to determine which microbial taxa moderated the positive impact of vaginal delivery on infant HCG trajectories, Cohen’s D effect sizes between vaginally delivered infants with AHCGT versus vaginally delivered infants with each of the three SHCGT groupings were calculated using R package rstatix version 0.7.0 without assuming equal variances, and microbial taxa that were consistently augmented or diminished in abundance across the three groupings were reported. Detailed statistics are provided for the significant microbial taxon abundances (Table S12).

The clinical factors examined included gestational age at birth, birthweight, birth head circumference, sex, total days of all antibiotics, longest number of consecutive days of all antibiotics, total amount of enteral feeds, total days of total parenteral nutrition, day of life full enteral feeds achieved, total amount of human milk, total amount of formula, and number of morbidities. Individual antibiotics or morbidities were not examined for this analysis, as some were too rare in incidence to be properly assessed. Cohen’s D effect sizes between infants with AHCGT versus any SHCGT were calculated separately for each delivery mode using R package rstatix without assuming equal variances. Detailed statistics are provided for the significant clinical factors (Table S8).

## Supplementary Material

Supplemental Material

## Data Availability

The data that support the findings of this study are openly available in NCBI SRA at https://www.ncbi.nlm.nih.gov/bioproject/PRJNA739139/. The authors would like to thank the Digestive Disease Research Core Center at the University of Chicago (P30DK42086) for its support on this study.
