# Supplementary material for: Bacteroidota and Lachnospiraceae integration into the gut microbiome at key time points in early life are linked to infant neurodevelopment
Source: Gut Microbes. 2021 Nov 28;13(1):1997560. doi: 10.1080/19490976.2021.1997560 (PMC8632288; doi:10.1080/19490976.2021.1997560)
Supplement: Supplemental Material [file KGMI_A_1997560_SM6479.zip › Supplementary information/Supplementary data caption revised version.docx]

Table S1. Non-significant differences in alpha diversity (richness and Shannon metric) evaluated by 16S rRNA gene sequencing of stool samples collected in the neonatal intensive care unit between infant head circumference growth (HCG) groups. Head circumference growth (HCG) group is defined by the difference in head circumference z-score from birth to 36 weeks postmenstrual age (PMA) as calculated by the Fenton growth curve: appropriate head circumference growth trajectory (≥0.5; AHCGT), mildly suboptimal head circumference growth trajectory (<0.5-1; mildly SHCGT), moderately suboptimal head circumference growth trajectory (<1-1.5; moderately SHCGT) and severely suboptimal head circumference growth trajectory (<1.5; severely SHCGT). Significance was determined by ANOVA on multivariate regression with HCG trajectory and PMA as fixed effects and patient as a random effect. The marginal coefficient of determination for generalized mixed-effect models (variance explained by fixed effects) is also reported as the R^2^.

Table S2. Games-Howell post-hoc analysis with pairwise Cohen’s D effect sizes of Welch’s ANOVA on difference in head circumference z-score from birth for each postmenstrual age (PMA) completed week between infant head circumference growth (HCG) groups. Head circumference growth groups are defined in legend for Table S1.

Table S3. Clinical characteristics of the Microbiome In Neonatal Development (MIND) cohort of infants at the University of Chicago Comer Children’s Hospital. Head circumference growth groups are defined in legend for Table S1. Binary variables are reported as the percentage of patients (number of patients) per group, with *p* values calculated by the Fisher’s exact test. Numerical variables are reported as the mean, variance, and standard deviation per group, with *p* values calculated by Welch’s ANOVA and Games-Howell post-hoc analysis, or alternatively pairwise Welch’s two/one sample t-tests, with pairwise Cohen’s D effect sizes.

Table S4. Prevalence of sub-taxa within each key microbial taxon depleted in the gut microbiome of infants with suboptimal head circumference growth trajectories during the examined completed weeks postmenstrual age (PMA) timeframes, with changes in the limited morbidity (LM) subset indicated in parentheses. Head circumference growth groups are defined in legend for Table S1. Only sub-taxa that were present in at least three patients of one study group are listed.

Table S5. KEGG pathways related to metabolism that contain the most significantly differentially abundant or prevalent KEGG database orthologies (KOs) between infants with appropriate head circumference growth trajectories (AHCGT) versus suboptimal head circumference growth trajectories (SHCGT) in the limited morbidity subset. Study groups defined by difference in head circumference z-score from birth to 36 weeks postmenstrual age as calculated by the Fenton growth curve: appropriate head circumference growth trajectory (≥0.5; AHCGT) and suboptimal head circumference growth trajectory (<0.5; SHCGT). For KEGG pathway classifications, the total number of significantly differentially abundant or prevalent KOs is indicated with at least 3 being requisite for listing. Significance for abundance was evaluated by ANOVA on multivariate regression with infant head circumference growth trajectory and postmenstrual age as fixed effects and patient as a random effect, and for prevalence by the Fisher’s exact test (*p*<0.05; FP<1%). The *p* values, least squares mean differences (diff) with 95% confidence intervals and R^2^ values (abundance); *p* values and percent prevalence per study group (prevalence) are reported for the KOs that are more abundant or prevalent amongst infants with AHCGT. Significance was found both overall and during the 31-36 completed weeks PMA time window. Abbreviations: ns = non-significant.

Table S6. Clinical characteristics for the limited morbidity subset of the Microbiome In Neonatal Development (MIND) cohort of infants at the University of Chicago Comer Children’s Hospital. Head circumference growth groups are defined in legend for Table S5. Binary variables are reported as the percentage of patients (number of patients) per group, with *p* values calculated by the Fisher’s exact test. Numerical variables are reported as the mean, variance, and standard deviation per group, with *p* values calculated by Welch’s t-test with Cohen’s D effect sizes.

Table S7. Details for the significantly differentially abundant fecal microbial taxa between vaginally delivered versus Caesarean-section delivered infants. Significance of taxon abundances was determined by ANOVA on multivariate regression with delivery mode and postmenstrual age (PMA) as the fixed effects and patient as a random effect; Benjamini-Hochberg (BH) adjusted values are additionally indicated. The marginal coefficient of determination for generalized mixed-effect models (variance explained by fixed effects) is also reported as the R^2^.

Table S8. Details for the significant moderating effects of clinical factors on the association between delivery mode and infant head circumference growth trajectory. Significance of moderation was determined by the Wald statistic on cumulative link mixed regression models for the interaction between delivery mode and the given clinical factor on the outcome of gradient head circumference growth trajectories as defined in legend for Table S1, with postmenstrual age (PMA), and delivery mode and the given clinical factor individually, as additional fixed effects plus patient as a random effect. McFadden’s R^2^ was calculated through dividing the log likelihood of the model by the log likelihood of the null model, and then subtracting this value by one.

Table S9. Details of redundancy analysis for evaluation of changes in β-diversity of the infant fecal microbiome over completed weeks postmenstrual age (PMA) by head circumference growth group. Head circumference growth groups are defined in legend for Table S1. Data is provided for both the complete dataset (all study groups) and limited morbidity subset (AHCGT versus any SHCGT).

Table S10. Details for the significantly differentially abundant or prevalent fecal microbial taxa and KEGG database orthologies (KOs) between infants with differing head circumference growth trajectories. Head circumference growth groups are defined in legend for Table S1. Significance of taxon or KO abundances was determined by ANOVA on multivariate regression with infant head circumference growth as the fixed effect and patient as a random effect; Benjamini-Hochberg (BH) adjusted values are additionally indicated. The marginal coefficient of determination for generalized mixed-effect models (variance explained by fixed effects) is also reported as the R^2^. Significance of microbial taxon or KO prevalence was evaluated by the Fisher’s exact test, with percentage of patients (number of patients) reported per group. Multivariate regression models were created for the postmenstrual age (PMA) time windows of 24-30 completed weeks and 31-36 completed weeks, in addition to overall (in this case adding PMA as an additional fixed effect). Significance was evaluated between all four study groups (all gr.) as well as AHCGT versus any SHCGT (2 gr.), and for both the complete dataset and limited morbidity subset. The specific linear mixed regression models used for generating study group least squares means for plotting in Figure 2 are provided at the end of the table.

Table S11. Details for the random forest classifiers predicting infant head circumference growth trajectories. Distinct random forest classifiers were built for each of the key time windows, 24-30 completed weeks postmenstrual age (PMA) and 31-36 completed weeks PMA, for predicting the binary head circumference growth trajectories defined in legend for Table S5. The variables used to build the models, out of bag (OOB) estimation of error rates, confusion matrices and tables of feature importance are provided. For feature importance, the mean decrease in Gini and parameters computed from permutation importance out of 1001 permutations are reported.

Table S12. Details for the significant moderating effects of fecal microbial taxon abundances on the association between delivery mode and infant head circumference growth trajectory. Significance of moderation was determined by the Wald statistic on cumulative link mixed regression models for the interaction between delivery mode and the given fecal microbial taxon abundance on the outcome of gradient head circumference growth trajectories as defined in legend for Table S1, with postmenstrual age (PMA), and delivery mode and the given fecal microbial taxon abundance individually, as additional fixed effects plus patient as a random effect. McFadden’s R^2^ was calculated through dividing the log likelihood of the model by the log likelihood of the null model, and then subtracting this value by one. The Cohen’s D effect sizes between vaginally delivered infants with AHCGT and vaginally delivered infants with each of the three categories of SHCGT are also provided for each microbial taxon.
